# Learnings from the United Kingdom infected blood inquiry

**DOI:** 10.1016/j.rpth.2024.102458

**Published:** 2024-05-23

**Authors:** Michael Makris, Brian O’Mahony

**Affiliations:** 1 School of Medicine and Population Health, University of Sheffield, Sheffield, United Kingdom; 2 Irish Haemophilia Society, Dublin, Ireland; 3 Trinity College Dublin, Dublin, Ireland

On May 20, 2024, Sir Brian Langstaff delivered the findings of the inquiry into the infection of thousands of persons with hemophilia with HIV and/or hepatitis C through the use of factor (F)VIII or FIX concentrate provided to them by the UK National Health Service [1]. This was the largest and longest public inquiry in UK history, lasting almost 6 years. The inquiry was called by Theresa May, the UK Prime Minister at the time, on July 11, 2017, and it commenced on July 2, 2018.

The infected blood inquiry (IBI) found there were systemic, collective, and individual failures to deal ethically, appropriately, or quickly with the risk of infections via blood or blood products or to act on a timely basis when the risk materialized. Among the many findings were failures to inform recipients of FVIII or FIX concentrate of the risk of transfusion-transmitted infections and failure to establish adequate mitigation measures to reduce the risk of HIV infection. The inquiry noted that many clinicians were reluctant to stop using factor concentrates or take measures such as reverting to cryoprecipitate or adjusting treatment regimes until the risk was clarified. The inquiry commented that a risk requires action and that certainty that the risk event will actually occur is not required. The IBI recommended that the UK Government provide compensation to infected individuals and the families of those who already died without further delay.

The risk of transfusion-transmitted hepatitis was well known in the 1970s, and using paid donors as well as pooling of plasma donations increased the risk. The lack of serious early problems from hepatitis led many clinicians to believe erroneously that this was a small price to pay for the ready availability of concentrates, which could be used at home for the rapid treatment of bleeds. Evidence presented to the inquiry suggested that this infective risk was not discussed with most patients. Plasma-derived concentrates were prepared from pools of 30,000 or more plasma donations. In the 1980s and 1990s, the risk of hepatitis C in UK volunteer blood/plasma donors was around 1 in 1500 [2], while in the United States, the paid donors included prisoners, among whom the hepatitis C virus rate was 15% [3]. With these donor hepatitis C virus infection rates, it can be appreciated that hepatitis C infection was almost universal after the first factor concentrate treatment prepared from pooled plasma, irrespective of whether this was prepared from voluntary or paid donors. A major difference was noted in the risk of HIV infection since UK products produced from voluntary donors carried a much lower risk due to the lower frequency of HIV in the United Kingdom in the early 1980s. The UK authorities were criticized for not investing in the expansion of the domestic fractionation service, as promised in the 1970s. Had this been done, the number of HIV-infected persons with hemophilia is likely to have been fewer, but this would have had a minimal effect on the number infected with hepatitis C. The report also criticized the failure to encourage or finance any research into viral inactivation by the UK fractionators, which could have led to a lower number of cases of hepatitis C infection and possibly a large reduction in infection with HIV if implemented early enough.

Much of the criticism centered around the response to the HIV epidemic. The initial alert came from the Centers for Disease Control in the United States on July 16, 1982, when they announced that 3 persons with hemophilia in the United States had AIDS. There were many initial uncertainties, but by March 1983, the Centers for Disease Control was sufficiently confident to issue a warning that blood products were responsible for AIDS in hemophilia. Despite this, in the United Kingdom, it continued to be stated that there was no strong evidence or certainty that HIV was a blood-borne virus. In this era, before the precautionary principle, action was often not taken until strong evidence was available. The proof, as it transpired, was the infection of thousands of people with hemophilia with HIV. The inquiry is very critical of this approach, stating that patient safety must be the guiding principle and that false reassurance was provided to patients and to the public. Possible measures to reduce the risk such as using cryoprecipitate or desmopressin instead of factor concentrate (especially for those with mild hemophilia), delaying elective surgery, using UK volunteer donor concentrates rather than US paid donor products, and limiting the number of batches individuals were exposed to were discussed at the time, but their implementation varied widely, and the result was significant differences in the proportion of persons with hemophilia infected with HIV in different hemophilia centers.

Despite the exhaustive nature of the IBI, several factors impacted its investigations, including the length of time since the events occurred, the nonavailability of the clinicians involved to give evidence (through death or illness), the death of patient organization leaders from HIV or hepatitis C, as well as the destruction of documentary evidence by the Department of Health, which is criticized as a deliberate attempt to make the truth more difficult to reveal. Although the IBI investigated UK events, similar issues were encountered in many countries where treatment of hemophilia was with factor concentrates. The way each country responded varied enormously, and in some, such as France and Japan, individual doctors were imprisoned. The fact that patients were infected in multiple countries to a similar level suggests that there was a systemic problem, but the extent of the problem and the responses of clinicians, patient organizations, and governments varied from country to country. The UK Government is criticized for falsely and repeatedly communicating that people with hemophilia had access to the best available treatments, infections were inadvertent, and screening for hepatitis C could not have been undertaken prior to September 1991, despite clear evidence to the contrary. It is greatly regretted that the UK inquiry took so long to be established, reporting 30 years after the US report [4], 27 years after Canada [5], and 23 years after Ireland [6]. This UK delay added to the distress and suffering in the community affected.

We conclude with learnings from the IBI and the response of the hemophilia community since the events examined over the past 40 years:

1. Transfusion-transmitted infection risk by concentrates can be eliminated with the use of recombinant products. Where plasma-derived concentrates continue to be used, well-established and effective viral inactivation techniques are now used.
2. Patients should expect their clinical team to discuss with them all the advantages and disadvantages of specific treatments not only when they are changing products but also while using the product if the evidence for efficacy or safety changes. Both known and unknown but potential risks should be discussed and documented.
3. Patients should seek multisource information about the relative benefits of the different treatments and should not rely on their clinical teams alone to provide them with information. Their representative patient organization should be resourced to play a role in patient education and empowerment.
4. Adequate counseling is essential before blood tests, and this should include information about how the result will be conveyed to the individual.
5. When patients are injured by their treatment, there should be early compensation without the need for litigation. The very lengthy delay in providing compensation from the UK Government exacerbated the suffering of individuals and families, and the compensation came too late for the thousands who had already died.
6. A statutory duty of candor should be introduced. An individual should not be blamed for “owning up” to a mistake, but blame should be apportioned if they stay silent. This also includes government and civil servants, where the culture of defensiveness and lack of transparency needs to change, and senior civil servants should be held accountable for the candor of advice they deliver to ministers.
7. Clinical audit should include measurement of patient satisfaction and concerns.

The inquiry report makes for sobering and sad reading. As we look at this in 2024, much of the clinical culture and practice criticized has evolved and changed from the paternalistic approach common 40 years ago, but the slow, inadequate, and incomplete response to this issue by the Government and others merits rapid deliberation of and action on the recommendations.
